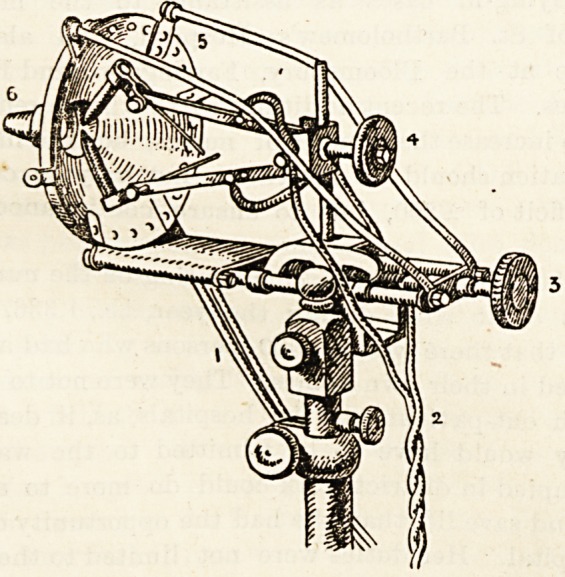# The Hospital. Nursing Section

**Published:** 1903-06-06

**Authors:** 


### The Hospital.

### Nursing Section.

Contributions for this Section of "THE HOSPITAL" should be addressed to the EDITOR, "THE HOSPITAL" NURSING SECTION, 28 & 29 Southampton Street, Strand, London, W.C.

No. 871.—VOL. XXXIV. *SATURDAY, JUNE* 6, 1903.

### Notes on News from the Nursing World.

### QUEEN ALEXANDRA'S ROYAL NAVAL NURSING SERVICE.

THE Queen presented badges on Friday last at Buckingham Palace to 24 sisters of Queen Alexandra's Royal Naval Nursing Service. Their names are as follows :—*Head Sisters*—Grace H. Mackay and Florence Cadenhead. *Nursing Sisters*—Mary H. O'Bryen, Louisa A. Fleming, Mary E. Hayes, Florence A. Moore, Florence H. Porter, Margaret H. Keenan, Isabel M. Beattie, Mary L. Grant, Mary C. H. Collings, Mary Bartlett, Rosa Northcote, Elizabeth E. M. Partridge, Kathleen Glasspoole, Mabel Beresford, Elizabeth Gratton, Mabel Basden-Smith, Alice Stanton, Ada E. Woodruff, Maud Phelan, Eleanor Veale, Florence Bates and Pickard.

### A NOVEL GUARD OF HONOUR.

THE visit of the King and Queen, the Prince and Princess of Wales, and other members of the Royal family to St. Paul's Cathedral on Sunday afternoon, will be of special interest to hospital nurses. The committee of the Hospital Sunday Fund have arranged that a number of nurses attached to the principal London hospitals shall be given the end seats right and left of the centre aisle in the nave down which the Royal party will pass to a position near the pulpit. The effect will be that the nurses, who will, of course, be in uniform, will constitute a guard of honour on the memorable occasion.

### NURSING QUESTIONS IN PARLIAMENT.

JUST before the House of Commons adjourned for the Whitsuntide recess Mr. Talbot asked the President of the Local Government Board whether he had taken into consideration the report of the Departmental Committee on Poor Law Nursing, dated November 10th, 1902, and whether it was still his intention to recommend the training of a class of qualified nurses in the smaller workhouses. Mr. Long's reply was that he could only state at present that the report referred to is under his consideration. We think that, seeing the report has been in his hands for more than six months, the President of the Local Government Board should have been able to give a definite answer to Mr. Talbot's question. Another topic of interest to nurses will come before Parliament on Tuesday next. Sir John Leng will ask the Secretary of State for War "whether he will explain why, seeing that the doctors, nurses, men, and orderlies serving in hospitals in South Africa have all been allowed hitherto to wear clasps with their medals, the privilege has been withdrawn from the nurses, and from them only." It is not conceivable that there is any desire on the part of the War Office to slight the members of the nursing profession ; but the explanation for which Sir John Leng asks is required.

### CENTRAL MIDWIVES BOARD.

AT a meeting of the Central Midwives Board on Thursday last week, a letter was read from the secretary of the Liverpool Ladies' Charity and Lying-in Hospital praying for the recognition of their certificate in midwifery as a sufficient qualification under Section 2 of the Midwives Act, 1902. The secretary was instructed to reply that the application would have the careful consideration of the board when they came to deal with the question of the acceptance of "other" certificates under Section 2. A draft form of notice of the effect of the Act, for the use of local supervising authorities was considered, amended, and approved. The secretary reported as to negotiations for acquiring suitable offices for the board, and further instructions were given to the sub-committee in charge of the matter. The secretary was instructed to prepare for the consideration of the board at their next meeting draft forms of midwives' roll and certificate under the Act.

### A GENEROUS GIFT TO A DEVONSHIRE PARISH.

AN interesting announcement was made by Sir William Walrond, M.P., Chancellor of the Duchy of Lancaster, at a friendly societies' *fête* at Uffculme on Monday. Sir William, who recently lost his son under sad circumstances, alluded to the value of good nursing, and after remarking that he knew the value of it in the case of his poor son, stated that in remembrance of the benefits he derived from it, Lady Walrond and himself would provide Uffculme with a nurse. We understand that the nurse will be at the disposal of all the parishioners, and that she will be installed as soon as Sir William and Lady Walrond have had an opportunity of discussing the details with the vicar of the parish and other residents.

### NURSES FOR IRISH CONGESTED DISTRICTS

THE appeal made by Lady Dudley a few months ago for assistance towards the establishment and maintenance of Jubilee nurses in some of the poorest and most congested districts in Ireland has met with a prompt response. Nearly a thousand pounds has already been received in donations and annual subscriptions, and Lady Dudley acknowledges particularly the kindness and co-operation of the Institute for Jubilee Nurses in London, who in addition to an annual subscription of £180—being the interest of the sum subscribed in Ireland towards the Women's Memorial to Queen Victoria—have rendered her much valuable help in carrying out her scheme. Lady Dudley is now in a position to make provision for three nurses in three of the most needy districts, and she hopes that two of them will begin work before the end of July.

### THE PROPOSED "NATIONAL TRAINING SCHOOL FOR MIDWIVES."

A meeting is to be held in London on the 18th of this month in order to ventilate the proposal for the formation of a National Training School for Midwives, to which reference was made in our issue of March 21st. Miss Alice Gregory, who is one of the promoters, attended a conference at Bristol the other day which had been convened to consider the future of district midwifery in England. In her speech, Miss Gregory, who has herself been working as a district midwife for seven years, alluded to the scheme, and mentioned Woolwich as a suitable place for the erection of a new general and maternity hospital. Miss Gregory, in replying to our criticism that it is not desirable to create yet another order of nurses, who, while highly trained for midwifery work, might, after their two years' course at the proposed training school, take up other branches for which they have not been adequately prepared, said it is obvious that "this risk is incurred by all the many hospitals who consent to take paying probationers for short periods." Whatever risks there may be at present, we do not desire to augment them. Moreover, it is quite practicable to give the best possible training to midwives in connection with the existing hospitals, some of which would be glad of a new source of income. In this way the expense of erecting, with all its risks, a brand-new institution where "educated women would receive an 18 months' course of general and monthly nursing, prior to a six months' course of midwifery in hospital and district," would be avoided.

### THE NORTH-EASTERN HOSPITAL FOR CHILDREN.

The new building which faces Hackney Road contains, besides wards for 54 beds, two sick-rooms for any of the nursing staff who may fall ill in the performance of their duties. Adjoining is a balcony which will be devoted to the use of the nurses, from which an extensive view is obtained. Included in the complete scheme which the committee of the North-Eastern Hospital for Children have in hand is a nurses' home, and when this is added, the houses rented for the nurses some little distance from the hospital will no longer be required for the purpose. The tram fares of the nursing staff to and from the home amounted during 1902 to £100.

### PHYSICAL TRAINING FOR NURSES.

The members of the Chelsea Infirmary Nurses' League assembled on Wednesday evening last week to listen to a lecture on "Physical Training as it Affects the Nurse." The lecture was delivered by Mr. Eustace Miles, the well-known athlete, and Miss Ina Stansfeld, one of the Local Government Board inspectors, presided. Several of the matrons of the metropolitan infirmaries were present, at the invitation of Miss Eleanor Barton. Miss Stansfeld said she hoped that the time would soon come when infirmary probationers would go through a preparatory course of physical instruction to enable them more readily to endure the strain of their work in the wards. She thought that drill and gymnastics should form a part of the curriculum in every metropolitan infirmary. For the sake of her patients a nurse should sacrifice some of her well-earned leisure, in order to make herself physically better fitted for her work. The lecture, which was closely followed by the nurses, was illustrated by some specimen exercises, and fencing was recommended as combining the needful movements. Ping-pong, lawn tennis, cricket, if properly adapted, and fives were also highly commended. Mr. Miles insisted on right methods of breathing, full muscular movements in both directions, absence of "grip" for the hands, relaxation, and exercises of the muscles of the trunk. He said that exercises, to be of use to the nurse, should involve the use of muscles not called into action in her work, and there should be absence of tension during rest. Exercise should not, he told the nurses in reply to questions, be taken after a long day's work, but before it began, and the clothing worn should be as light as possible.

### DISTRICT NURSING IN DERBYSHIRE.

Lady Laura Ridding, wife of the Bishop of Southwell, who presided at the annual meeting of the Derbyshire County Nursing Association last week, replied to those who had criticised the action of the Association in becoming affiliated with the Queen Victoria Jubilee Institute. She pointed out the importance of having properly-trained nurses, under the supervision of a nurse inspector, and contended that the advance made during the past year had fully justified the steps taken, seven new districts having been worked. Some people, she said, implied that the Association was in opposition to the Derby Infirmary. On the contrary, it assisted the infirmary, because many of the patients attended by the nurses would otherwise have to go there and occupy beds which are used for more serious cases. In the course of the annual report it was stated that out of 74 districts in the county with a population of over 1,000 only 20 had nurses working in them. The extension of the work was, however, hampered by lack of funds, and the committee suggested that meetings should be held in various districts with the object of arousing interest in the matter.

### GRANARD GUARDIANS AND NUNS.

At the meeting of the Granard Guardians last week two nursing nuns tendered their resignation by direction of their ecclesiastical superiors. Mr. Masterton moved that the Board refuse to accept the resignations and pledge themselves to support the nuns. This motion was carried unanimously, and a deputation was at once sent to the Rev. Mother, and reported on its return that the nuns complained of Dr. Kenny's bearing to them since the inquiry. The Rev. Mother said she would consent to allow the sisters to remain only on condition "that the Guardians would insist upon the doctor not using insulting language towards them." The report says that "a motion to this effect was passed," but so long as the nature of the alleged insulting language is not disclosed, Dr. Kenny should certainly not be condemned. The Granard Guardians next called upon the Irish Local Government Board to "inquire into the want of harmony between Dr. Kenny and the nursing staff," a request, we think, not likely to be complied with.

### CHRISTCHURCH WORKHOUSE INFIRMARY.

The Local Government Board having informed the Christchurch Guardians that their sanction is not necessary to the appointment of a medical man to lecture to the probationers at the workhouse infirmary, the Guardians have arranged with Dr. A. H. B. Hartford to deliver a course of lectures extending over a period of six months. The fee, half a guinea per week, will not ruin the ratepayers. Changes have also been made in the nursing arrangements of the infirmary. In future a charge nurse is always to be on duty, and the probationers are to take night duty in rotation. There will thus be a probationer as well as a charge nurse regularly on duty at night.

### THE VISITING NURSE.

At a meeting of the Marylebone Daily Visiting Nursing Association held last month the Dowager Lady Desart, the honorary secretary of the organisation, stated that it was formed 14 months ago, and that it was hoped that it would eventually be self-supporting. In the meantime the committee appealed for increased subscriptions and donations. This is a very unsatisfactory state of affairs. The charges made for the visiting nurse of the Association are 2s. 6d. for a visit of one hour, 12s. 6d. a week for one daily visit, and 18s. 6d. a week for two daily visits. In no sense, therefore, can it be called a charity, and we do not think that the public can be expected to support it after the initial stage has been passed. The idea of a visiting nurse for those who cannot afford to pay for the exclusive services, and are yet not suitable patients for the district nurse, is an excellent one. But it must be carried into effect on business principles.

### COTTAGE NURSING.

The tenth annual conference of Affiliated Benefit Nursing Associations for the Supply of Cottage Nurses on the Holt-Ockley system was held at 48 Eaton Square last week. Miss Broadwood, the honorary secretary, read the report, which showed that during the year 75 nurses were sent into training and placed on the Cottage Nurses' Register. There are now 138 affiliated and about 79 non-affiliated benefit nursing associations. The expenses in connection with the work for the year were £343, while the income was only £262, leaving a deficit of about £80. Lord Ancaster said that the society had been doing good work and extending its operations in different parts of the country. With the extension of operations there must be more work at headquarters, and if that was not properly carried out the functions of the society must suffer. Mrs. Heywood Johnstone gave an account of her work in connection with the Midwives Act and the establishment of the Rural Midwives' Association. Lady Ancaster asked that the affiliated associations should pay a small extra sum annually. Mrs. Edward Huth having addressed the conference and advocated that the associations should contribute 2½ per cent. of their income, Miss Whitmore Jones spoke on the subject of the establishment and working of groups for lending nurses, after which Dr. Robert Boxall addressed the conference on the Midwives Act and its effect on benefit nursing associations.

### THE HAHNEMANN HOSPITAL, LIVERPOOL.

A new Nurses' Home and Laundry in connection with the Hahnemann Hospital, at Liverpool, has just been opened. The home is built apart from the hospital, from which it is approached by a covered passage. The dining-room is painted crimson, and the sitting-room a delicate blue, the carpets and curtains being in harmony. Handsome engravings adorn the walls, and a piano is one of the most welcome features. The bedrooms are pretty and cheerful. The Lady Mayoress, at the opening ceremony, which was remarkable for the profusion of flowers, plants, and palms, wished the nurses all success, and afterwards, in company with the matron and the guests, proceeded on a tour of inspection. The idea of a home and laundry originated with Lord Dysart, who offered to contribute £500 if the rest of the money required could be obtained within a given time.

### BAZAAR AT NORTHAMPTON.

A very successful bazaar was held last week at the Victoria Nurses' Home, Northampton. As a result, the sum of £35 has been paid to the District Nursing Fund. Much of the organisation of the bazaar was carried out by Miss Lunn, superintendent of the home, who was assisted by all the nurses, and the various useful and pretty articles offered at the stalls were mainly the work of the Queen's Nurses' Needlework Guild.

### WOBURN COTTAGE HOSPITAL.

The nursing staff at the Woburn Cottage Hospital, the spacious new building which the townspeople owe to the munificence of the Duchess of Bedford, consists of the matron, Miss A. Young, two nurses, trained at the London Hospital, and a probationer. The accommodation is for four men, four women, and two children, and ample nursing provision has therefore been made for the patients. The comfort of the nurses themselves has been most carefully studied. They have a dining-room facing the surgery, a pretty drawing-room with a piano, a sofa, easy chairs, and other accessories. There is a nurses' kitchen where the food is prepared for the patients under the direct supervision of the matron, the household staff supplying the wants of the nurses and servants in another kitchen.

### SHORT ITEMS.

The Princess Louise has promised to attend the Battersea Town Hall, on June 30, for the purpose of receiving purses on behalf of the Surrey District Nursing Association.—The s.s. *Guelph*, which arrived from South Africa on Sunday, had on board Nursing Sisters MacLean, McVean, and I. Maxwell.—Another small gathering for district nurses was held at Selworthy, Somerset, last week. The district is scattered, and it is difficult to collect the nurses from the various villages, but those who were present had a pleasant afternoon. A paper was read on "Infant Feeding," and was followed by some discussion.—Two charge nurses of the Middlesbrough Union Infirmary, Misses C. M. Montague and H. M. Carling have obtained the certificate of the London Obstetrical Society. Miss T. Imeson, who recently finished her three years' training, has also obtained the same certificate.

### The Nursing Outlook.

"From magnanimity, all fear above;
From nobler recompense, above applause,
Which owes to man's short outlook all its charm."

### NUNS AS NURSES.

The "surprise" of the Irish Local Government Board at discovering "the list of nursing duties that the nuns of the Granard Workhouse gave as being outside their province as nurses in charge of sick wards" shows once more the weird innocence and sleepy backwardness apt to fall on Government departments. For surely the fight which is barely over in Paris, the fight which is going on almost daily in Ireland, must be known to all interested in the care of the sick. It is the fight as to whether the nun with a vocation can also be a nurse with a profession; whether the vows of the religious are not incompatible with the necessary obedience of the nurse to the doctor. It ought not to be so in theory when one remembers the celebrated words of St. Vincent de Paul in founding the order that bears his name—"Your convent must be the house of the sick; your cell, the chamber of the suffering; your chapel, the parish church; your cloister, the streets of the city or the wards of the hospital; your rule, the general vow of obedience; your grille, the fear of God; and your veil, your modesty." That was in 1634, and yet in the beginning of the twentieth century the nuns have not yet learnt to put their own elaborate rules aside and take the one simple one—

"All for Thy sake, Lord: I will see
In every sufferer—Thee!"

The result is that they are gradually being ousted from the highest form of charity; that the work of tendance on the suffering which was one of the chief reasons and the best apology for the religious orders in the Middle Ages is passing from their hands, and we cannot believe but that the spiritual side of a sister's life must be often injured and warped when this most Christ-like form of service is taken from her. And it is taken from her because she fails to recognise that Christ not only healed the sick, but that He washed the feet of His disciples; if the nuns will not wash the feet—will not do the lowest and most humble deeds—neither will they heal the sick nor save souls. Surely in the great revival of the religious spirit, particularly of the order of St. Francis, someone may be found who will reform the nursing orders of the Roman Catholic Church, put them more into touch with the needs of the day, and breathe into them the spirit of humility and self-sacrifice which is the breath of life for all religions.

Unless this is done—unless the nun will become more nurse-like, will wear cleanly and suitable garb, will perform all ordinary nursing duties however dirty or disagreeable, will say her prayers "inwardly" in the ward when necessary, instead of going to chapel and leaving the patients in pain; will carry out the doctor's orders faithfully as part of her duty, she will have to be dismissed from the hospital and the infirmary. This would indeed be a disgrace to those who through many dark and dreary days of the past upheld the service of the suffering. We cannot but remember what nursing owes to the religious orders; we cannot but remember how in Dr. Gasquet's book "The Great Pestilence" there are continual quotations from authorities who state "charity was dead," "even the doctors did not dare to visit the sick," and so on, and that the only instance given of nurses true to their post is the nuns of the Hôtel Dieu of Paris, thus mentioned in the chronicle of William of Wangis—"So great was the mortality in the Hôtel Dieu of Paris that for a long time more than 50 corpses were carried away each day. And the devout sisters, not fearing death, worked piously and humbly, not out of regard for any worldly honour. A great number of these sisters were summoned to their reward by death and rest in peace with Christ, as is piously believed."

And here is an incident from the history of the same order, which shows how in the past nuns did not scorn to change with the times and to put before all else their service to the sick. In the crash of the French Revolution all religious institutions were dissolved; yet in 1801 a remarkable consular order was issued empowering "Citoyenne Duleau, formerly Superior of the Sisters of Charity," to train young women for hospital work. This was the method of securing the continuance of their work a hundred years ago. Surely, if the nuns could bow to such necessity then, they might now reform and improve their regulations and save us from any more scandals in Irish workhouse infirmaries. Finally, we cannot but remember that when Florence Nightingale went to Kaiserwerth she found that the deaconesses had derived part of their rule, and even their dress, from the Dames of St. Augustine; and that when she went to Paris she studied under the very Sisters of Charity of whom we have been writing. Nurses of to-day owe much to the nuns of yesterday; and yet we have no hesitation in saying that the nun of yesterday has no right to be a nurse of to-day. Even a nun must move with the times if she would work as well as pray; and if her work is in the wards she will soon discover the great truth that life can be as strictly disciplined outside a convent as inside.

### Lectures on Medical and Surgical Nursing.

By John Hopkins, F.R.C.S., Medical Superintendent of the Central London Sick Asylum District Asylums, Cleveland Street, W., and Hendon, N.W.

### LECTURE IX.—DISEASES OF NERVOUS SYSTEM—

(*Continued from page* 102).

*Meningitis*—Inflammation of the covering membranes of the brain and spinal cord gives rise to the effusion of inflammatory products, lymph which coagulates and ultimately organizes and fluid serum. This effusion compresses the contained nerve-structures and interferes with their function. In acute cerebral meningitis, the compression of the brain leads to coma. In chronic spinal meningitis, the lymph as it organises and contracts causes pain by pressing upon the sensory nerve-roots, and paralyses by compressing the motor-roots. This is known as *pachymeningitis.*

The inflammation, if at all acute, affects the nerve substance as well as its coverings, so that sensation becomes exaggerated. Light and sound may be unbearable, and simple touch may cause pain. This is known as hyperæsthesia. When there is inflammation anywhere in the body, the nerves in the inflamed part become over-sensitive, as we all know from personal experience. When the nerve-trunks are inflamed, the parts to which they are distributed become at first hyperæsthetic, but afterwards, if the nerves waste, as they often do, power of feeling is lost, and the parts so affected are then said to be anæsthetic. Power of movement becomes lost in like manner. When the nerves of the periphery (of the arms and legs) are inflamed, the disease is known as peripheral neuritis.

Irritation of a nerve causes severe pain sometimes. Most people know what the sudden onset of toothache is like. This is due to irritation of the bare end of a nerve exposed in the carious tooth. If this irritation be kept up, the pain is felt in the next teeth, then in the other jaw too, and lastly in other nerves that do not go to the jaw, but are distributed to the face and scalp. The patient is then said to have neuralgia. Much of the neuralgia experienced in the face and scalp has its origin in diseased teeth. Sometimes this can be distinctly traced, but often the patient will deny all irritation about the teeth; for the neuralgia may conceal the pain at the original source of the trouble.

In dealing with irritation and inflammation in the nervous system, the object must be to get rid of the source of irritation if possible, and to quiet and soothe the parts affected. When there is cerebral meningitis all the nerves of the body including those of the special senses are over-sensitive, so bright lights must be avoided, no noise must be made, and the patient must be disturbed as little as possible. When a nerve is inflamed the skin to which it is distributed needs protection; sometimes the passage of a current of cold air over the part is acutely painful, and this requires the protection of some soothing powder and a soft covering of cotton-wool.

The effects of inflammation are to temporarily paralyse parts. As the inflammatory products become absorbed there is a tendency for nerves to regenerate. But it takes a long time for this to take place, and in the meantime joints become stiff, and muscles waste, and when the nerves are restored the muscles cannot bend the joints. This might lead to a patient becoming chronically bedridden. It may be avoided by keeping the parts in readiness against the time that the nerves will be restored. Passive exercise of muscles and joints in a regular and systematic manner then is the thing to be done. The limbs must be rubbed and the joints bent daily. Spasmodic affections of the nervo-muscular system assume many forms. Colic is the painful spasm of involuntary muscular fibre, whether in bowel, bile duct, ureter or uterus. Cramp is a painful spasm of voluntary muscle. Writer's cramp is a spasm of the muscles used in writing. Excessive use of muscles excites this spasm.

When the muscles are tightly contracted and rigid the spasm is called tonic, when they are alternately contracted and relaxed there is clonic spasm. They are both due to the same cause, namely, discharge of nervous impulse from grey matter along the nerves in successive waves. When the waves succeed each other very quickly the muscles are kept in a tonic state. This happens at the onset of an *epileptic fit*, but as the grey matter becomes exhausted the impulses follow one another less quickly and each spasm is followed by a relaxation of the muscles. With further exhaustion of nervous energy the clonic spasms become slower and finally cease. In dealing with a case of epilepsy it must be borne in mind that when the whole body is stiff, the muscles of the chest impede respiration, the lungs cannot expand blood cannot pass through the capillaries of the lungs in the absence of oxygen, the arteries being elastic continue to pour blood into the veins, which thus become over-distended, turn the face blue, and cause this and the neck to swell up and become turgid. It is at the onset of the fit, then, that the collar must be unloosed. When the clonic spasms begin the jaw and tongue begin to work, and the latter may be thrust out and get nipped and bitten by the teeth. The nurse then must be in readiness to thrust something between the teeth as soon as the jaw begins to move. There is no occasion to try to force it in whilst the jaws are spasmodically closed. When a fit is over the patient generally falls asleep. His nervous system is exhausted and needs recuperation. Epileptic fits vary in character. When there are convulsions the condition is called "grand mal," and "petit mal" when the fit is so slight that there are no convulsions, merely a passing giddiness perhaps. Epilepsy is due to a morbid discharge of nerve energy by the motor nerve centres. This constitutes the typical epileptic fit. Sometimes, however, the sensory nerve centres are the parts principally affected, and then there is a loss of normal consciousness. The patient passes into a state that resembles sleep walking, and the nervous discharges are more or less orderly instead of being chaotic as in the typical fit. He moves and speaks mechanically and may perform a variety of movements, such as walking, poking the fire, and putting on his coat and hat; or he may be seized with an impulse to perform some act of violence. When the attack has passed off, he remembers nothing of what he has done.

*Mental Diseases.*—The common type of this class found in infirmaries in considerable numbers is that of failing memory due to advancing years. This senile dementia betrays itself by steady loss of memory. The time of day, the day of the week, the month and the year are forgotten. The patient forgets where he is. He has little inclination to do anything and objects to be disturbed. There is a tendency to incontinence of urine, which is in part due to lack of inclination to move, but there is also failure to appreciate the situation; the mind does not awake to the occasion. A little assistance from the nurse, a thoughtful reminder, will do much in an early stage to help the patient back to a normal state. Occupation will restore these cases for a time. If the patient be aroused, and made to get up and occupy himself about the ward helping the nurse, a sufficient interest in life returns to keep him going. A waxy transparency of skin and disappearance of wrinkles is frequently seen in this class of disease, that gives to the patient an appearance of youthful simplicity so characteristic that the mental condition is at once suspected on seeing the case. As the disease advances there are signs of increasing irritability, and the patient may be abusive and resist any interference with him. Sometimes there are hallucinations of sight and of hearing, and distinct delusions based upon these. In gait the patient moves stiffly with short, shuffling steps, and after long confinement to bed the joints grow stiff. Superficial bedsores are likely to occur in the cases that are bedridden unless care is taken, and towards the last the deep sloughing variety of bedsore is sometimes seen. The same condition of mind is pretty frequently observed in old cases of hemiplegia, and there is the same tendency to deep bedsores towards the end of the case.

Delusions are not unfrequently seen in many different diseases. Especially are some heart and aneurism cases subject to them, as a result of disturbance in the regular supply of well aerated blood to the brain. Short of delusion there will be seen sometimes a disposition to suspicion, jealousy and quarrelling, a condition that the nurse should recognise to be a part of the disease. If fully aware of this she does not let her feelings be too readily played upon by the patient's behaviour. Patients with failing minds sometimes show a tendency to collect and to hoard things up. They will gather all the scraps from the plates after dinner and beg food from their fellow patients. Such cases will sometimes do this from having a morbid craving for food. They eat enormously and grow fat, and may be so ravenous that they will fill their mouths too full and may even choke. In such cases it is a nurse's duty to see not only that their patients get enough food, but also that they do not get too much.

### The Finsen Light Treatment.

BY HONNOR MORTEN.

(*Concluded from page 119.*)

### III.—NEWER LAMPS AND LATEST METHODS.

The illustration this week is of what is generally called the "French" lamp, and is a modification of the lamp invented by Lortet and Genoud, by the engineer of the London Hospital. It is an arc lamp with the two carbon points behind a metal screen (5) which is also a water-jacket; the points join just behind a perforation in the centre of the screen, and the light is focussed down the small tube (6). The screw for approximating the electrodes to the compressor at the end of tube (6) is marked (3); the screw, the nearing, and separating carbons is marked (4); the flexible wires conducting the current are marked (2); and the tubes for circulating the water are marked (1).

This lamp has these advantages over the big Finsen light:—

(1) The patient sits directly up to the lamp with the lens in direct contact with the skin; thus the compressor is done away with.

(2) It is much less costly to install and less costly to work.

(3) The patients being so much nearer to the light the séance only takes 15 minutes.

(4) The lamp is simple, the nurse can see every part of it; it is adjusted by the nurse by the screw B, and is kept at an amperage of 14.

So here we have a lamp only costing £25 to install and about 9d. an hour (one Board of Trade unit) to work, and which can treat four patients to the hour. It sounds delightful; are the results as good? Some writers say "yes," but those who have worked longest with the French lamp admit that the quicker the treatment the more likely the healing to break down, and the less pliable and flat the cicatrices. This lamp then, though excellent for slight cases, and where there is good healing power, is not the best in all cases. So far as nursing goes, the nurse is employed in adjusting the amperage, while the patient himself by pressing on A produces the necessary local anæmia.

But there is yet another lamp, constructed by Dr. S. Bang, Dr. Finsen's assistant, and having iron instead of carbon electrodes. Coolness is obtained by running water through the electrodes. This lamp is a quarter the size of the French lamp, a quarter the cost, needs only five ampères current, and will in five minutes produce sufficient reaction of the skin. This Bang's lamp was brought over last autumn by Queen Alexandra and has just been fitted in the new light department of the London Hospital. But it has already been at work in Copenhagen and Paris, and, exactly as might be expected, it intensifies the faults of the French lamp. That is to say that there are few cases that can stand such rapid treatment, and that the healing though swift is not nearly so certain. The old "tortoise" lamp is likely to prove the best in all cases, though the younger "hare" lamps may be most useful adjuncts. No doubt need exist that the light department which has all sorts of lamps is the best for all sorts of cases.

Here in England we have not enough lamps to treat the number of lupus cases calling for treatment, and so there have been but few experiments with other cases; but at Copenhagen and Paris acne, rodent ulcer, epithelioma and other diseases have been treated with varying results. There can be no doubt that we are only at the first stages of light therapeutics, and as they are founded on thoroughly hygienic principles, they will probably supersede serum therapeutics in many cases. Also exactly as there is a tendency to overdo the "open-air" cure with enthusiasts, so there will probably be a tendency to overdo the light cure, and to try and count it a specific for every ill.

With the $x$-rays treatment for chronic ulcers, sinuses and other ills, the nurse has seldom much to do, the apparatus being managed by the electrician. With the new electric lamps for giving light and vapour baths the nurse will probably have much to do, and will find them far easier and pleasanter than the clumsy vapour baths of old.

### Everybody's Opinion.

[Correspondence on all subjects is invited, but we cannot in any way be responsible for the opinions expressed by our correspondents. No communication can be entertained if the name and address of the correspondent are not given as a guarantee of good faith, but not necessarily for publication. All correspondents should write on one side of the paper only.]

### THE ONE-YEAR NURSE.

"Miss J. Wilson," Treasurer of the Workhouse Nursing Association, writes: May I answer as briefly as possible some of the points raised by Mrs. Richmond, matron of the Luton Workhouse? The preponderance of opinion against the one-year nurse is expressed by such authorities as the secretary of the Nightingale Fund, a fund to which advanced Poor-Law nursing owes a debt that should never be forgotten; by the matrons of the principal hospitals and infirmaries who have themselves been instrumental in providing nurses for workhouses; and by medical men, some closely associated with the poor-law, both as examiners and medical superintendents. If such a mass of opinion is clearly against the creation of the one-year nurse it is worthy of attention. Mrs. Richmond proceeds to say that those who express this opinion were not able to place before the Departmental Committee a practical scheme for providing a staff of nurses to the smaller workhouses, but if the minutes of evidence are consulted, it will be found that a scheme has been suggested for the formation of a nursing department of the Local Government Board, on which nursing experts should serve. This was proposed as far back as 1896 by this Association, it was urged by witnesses representing various shades of opinion, and it was supported by such authorities as Miss Gibson, matron of the Birmingham Infirmary; Mrs. Wates, matron of Lewisham Infirmary; Dr. N. Raw, medical superintendent of the West Derby Union; and Mr. Sydney Holland, chairman of the London Hospital and the Poplar Hospital, all having practical knowledge of the question of training nurses. Such a scheme implies a national system, organised for the purpose of aiding Boards of Guardians. It would arrange for the engagement, binding, training, and supply to smaller workhouses of suitable women, who should receive their first knowledge of nursing in a large infirmary or hospital, in which care of the sick is the first consideration, and in which training can be well organised. Those who support it have not been given the opportunity of hearing the objections to their scheme, nor have they been encouraged to work it out in detail. They can only regard any plan which aims at a short and insufficient training as merely a "specific" which will end in doing widespread harm, although it may for a time supply half-trained nurses to the smaller workhouses. Further, to take Mrs. Richmond's arguments in order as they stand in reference to our Memorial. (B and C.) Our Association has had experience in the attempt to train in smaller workhouses; although we took every precaution the plan was not successful and had to be abandoned. The probationers had to unlearn much of what they had learnt in the small workhouses, although in each case they worked under a trained nurse. The drawbacks to creating two grades of nurses are to my mind made clearer than before by the very arguments used by Mrs. Richmond in defending the proposed plan. It should be always remembered that the one-year nurse will hold a certificate signed by three persons, one a doctor, and she is, therefore, in a different position from the nurses who have received "no systematic instruction whatever, but simply have a smattering of knowledge picked up here and there." No syllabus issued by the Local Government Board can make up for the lack of a sufficient number of varied cases, the careful observation of which forms the most important part of a nurse's training. It should also be noted that no remuneration is proposed for this extra work which, if done thoroughly, will occupy considerable time, and it is a question if a busy medical man could lecture regularly to one or two probationers. Is it not also obvious that the experience of matrons who do train thoroughly in larger institutions gives them a clear conception of the drawbacks and difficulties of small workhouses in attempting such work? In dealing with question (D) Mrs. Richmond proposes that on a medical man should be laid the responsibility of saying whether a patient requires "the skill of a one-year or a three-year nurse, and can state plainly which it is necessary for the patient to have." Diagnosis would be a much simpler matter than it is if illness ran constantly in the direction desired, but how frequently does influenza develop into pneumonia, apparently intestinal disturbances turn into typhoid, and is not the development of early symptoms the very point in which a nurse can be invaluable to the doctor and patient? Truly one more anxiety will be added to illness if, when a more serious complication is announced by the doctor, comes also the information to the patient and friends that the nurse must leave because she is only a one-year nurse, and is not therefore sufficiently experienced to go on with the case.

### Metropolitan Nursing Association.

By permission of the Duke of Westminster, the twenty-seventh annual meeting of the Metropolitan Nursing Association was held on Wednesday (last week), in the "Reuben's Room," at Grosvenor House.

The chair was taken by Sir William Broadbent, who was supported by Mr. H. Bonham Carter, the Rev. Dacre Craven Mr. F. D. Mocatta, Mr. C. S. Loch, and Dr. Oswald Browne.

The report, read by the Rev. Dacre Craven, stated that 15 nurse probationers had been admitted during the year, making, with eight already in training, a total of 23. Of these, 15 completed six months' district training, and eight remained at the end of the year.

Lectures were given on the "Diseases of Women," by Miss Appell, M.D.; on "Hygiene," by Mrs. Goslett; and on "Sick Cookery," by the Staff Teacher at the National School of Cookery. By arrangement with the authorities, nurses attended lying-in cases as assistants to the maternity students of St. Bartholomew's Hospital, and also gave attendance at the Bloomsbury, Farringdon, and Finsbury dispensaries. The recent additions to the home rendered it possible to increase the number of nurses, but the income of the Association should be increased, not only to cover the present deficit of £200, but to ensure continuance of the work.

Sir William Broadbent, in commenting on the number of new cases dealt with during the year, *i.e.*, 1,336, said he calculated that there were 50,000 persons who had annually to be nursed in their own homes. They were not to be compared with out-patients at the hospitals, as, if dealt with there, they would have to be admitted to the wards. A nurse occupied in district work could do more to alleviate suffering and save life than she had the opportunity of doing in the hospital. Her duties were not limited to the care of the patient, since she was responsible for the cleanliness of the sick room, and her influence in the homes she visited tended to a higher standard of sanitation and health. He could imagine nothing more discouraging than the life of a district nurse living an isolated life; this difficulty was, however, avoided in a district nursing home, where interchange of thought and common interests were possible. That so much had been done by the nurses for the sick poor on only £1,600 was, in his opinion, very wonderful. The cost of the same patients if treated in hospitals would have been much greater.

Mr. C. S. Loch, in moving a resolution pledging the meeting to support the association, described it as a preventive and remedial charity. By means of co-operation between hospitals, nurses, the clergy, and other workers, he thought that forms of relief which did nothing to improve character would gradually fall into discredit.

### Appointments.

Almondsbury Memorial Institute and Hospital, Bristol.—Miss Derrick has been appointed matron. She was trained at St. Bartholomew's Hospital, and has since been on the staff of the New Hospital for Women, London.

Birkdale Hospital for Infectious Diseases.—Miss Edith Owen has been appointed nurse-matron. She was trained at the Carmarthenshire and Anglesey Infirmary, Bangor, North Wales, and afterwards held posts at the St. Helen's Hospitals and the Royal Infirmary, Lancaster.

City Hospital, Seacroft, Leeds.—Miss Lily Abson has been appointed sister. She was trained at Bradford Union Infirmary, and has since been staff nurse at Warrington General Infirmary, Wakefield Union Infirmary, and sister at Woolwich Union Infirmary. She holds the L.O.S. certificate.

Colne Jubilee Cottage Hospital.—Miss M. J. McRae has been appointed matron. She was trained at the Royal Albert Edward Infirmary, Wigan, and has since been night sister at Grimsby and District Hospital, assistant matron at Cork Street Fever Hospital, Dublin, and district nurse at Newbury. She holds the dispensary certificate of the Apothecaries' Hall, London.

Corbett Hospital, Stourbridge.—Miss Katie Warburton has been appointed matron. She was trained at the General Hospital, Birmingham, where she has since been sister in charge of both male and female surgical wards.

Derbyshire Hospital for Sick Children.—Miss Mary Emily Thorp has been appointed matron. She was trained at the Royal Infirmary, Derby, where she was afterwards sister. She has also been sister in charge of a surgical home at Croydon, and ward sister and housekeeper at the Hospital for Sick Children, Great Ormond Street, Bloomsbury. She holds the L.O.S. certificate.

East Sussex Hospital, Hastings.—Miss May E. Moore has been appointed matron. She was trained at St. Mary's Hospital, Paddington, and has since been charge nurse at Meath Home for Epileptics at Godalming, sister at Sheffield Royal Infirmary, night superintendent at Leicester Infirmary, matron at the International Hospital, Naples, and Matron of a sanatorium in Switzerland. She holds the L.O.S. certificate.

North-Eastern Hospital for Children.—Miss A. M. Bushby has been appointed matron and Miss Scott-Cavell home sister. Miss Bushby was trained at the East London Hospital for Children and at King's College Hospital, subsequently returning to Shadwell, where she remained for ten years as sister and assistant matron. For two years and a half she has held the post of matron at the Isolation Hospital, Southampton. Miss Scott-Cavell was trained at the Metropolitan Hospital, Kingsland Road, and has since been engaged in nursing at the Hospital for Women, Liverpool.

Queen Victoria Jubilee Nursing Institute.—Miss Georgina B. Gale has been appointed Queen's Probationer at the St. Patrick's Nurses' Home, Dublin. She was trained for three years at the Wolverhampton and Staffordshire General Hospital.

Sheffield Royal Hospital.—Miss E. R. Tait has been appointed sister. She was trained at Liverpool Royal Infirmary, and was afterwards staff nurse in the same institution. She has since been sister at the Liverpool Skin and Cancer Hospital, has served for two and a half years as a member of the Army Nursing Service Reserve in South Africa, and been sister at the Bristol Royal Infirmary.

---

Bromsgrove Workhouse Infirmary.—With regard to the appointment of Nurse Clements as charge nurse at Bromsgrove Workhouse Infirmary, the clerk to the Bromsgrove Union states that she was not trained at St. Giles's Infirmary, Camberwell, but only held the post of ward nurse at Constance Road, Camberwell, where she took her L.O.S. certificate.

### For Reading to the Sick.

The Father is adored in prayer continually, and especially in the Lord's Prayer. The Son is worshipped in prayer and thanksgiving, especially at the close of every prayer in His Name. I must worship the Holy Ghost by prayer, by reverent thoughts, by loving and humble thankfulness, for His love. He is in direct touch with all creation. All created things are, in a special sense, His work. He is "the Lord and Giver of life."

All the fair order or *cosmos* of the natural world, its beauty, and glory, and sweetness, are brought out by Him. He is thus the Agent for carrying out, in creatures, the Eternal Will of God the Father and God the Son. He perfects and adorns in the spiritual world. The blessed angels feel his touch, and receive through Him their beauty. He brought before the angels the revelation of the Incarnation to lead them on to full blessedness. Those who refused fell, notwithstanding His loving Will; He led on to perfection those who embraced it. He is the Worker Who perfects Divine life in the souls of men. By many energies of Love He guides, comforts, warns, teaches, takes of the things of Christ, and shows them to souls.

*Canon Knox-Little.*

---

"The apostles lived in a vivid sense of experienced intercourse, first with the Son, then with the Father, through the Son, later with the Holy Ghost, and with the Father and the Son through the Holy Ghost. This vivid experience, outward and inward, made logical formulas unnecessary. When the formula of the Trinity—three Persons in one Substance—was developed in the Church later on, through the cross-questioning of heresies, it was with many apologies for the inadequacy of human language, and with a deep sense of the inscrutableness of God. The formula was simply intended to express and guard the realities disclosed in the Person of Jesus Christ, and great stress was laid on the divine unity."—*Gore.*

---

How wonderful! how beautiful!
The sight of Thee must be—
Thine endless wisdom, boundless power,
And awful purity.—*Faber.*

---

Whilst clearly proclaiming the unity of God, Jesus Christ revealed Himself as God's equal, and spoke of the Spirit whom He was about to send from the Father, as a divine Person. He declared that it was expedient, or advantageous, that He should depart, in order that the Spirit might come; and thus implied that the Spirit was His equal. Christ's departure could not have been expedient, unless the Spirit was divine as He was. He commanded His apostles, and their successors, to baptize all nations "into the name of the Father and of the Son and of the Holy Ghost." In this command, He summed up the doctrine of the unity of God in a trinity of Persons. Thus, Christ's language about Himself, the Father, and the Spirit, is the basis of the Christian belief that there is one God in three Persons.

*Vernon Staley.*

---

God the Father give us Grace
To walk in the Light of Jesus' Face.
God the Son give us a part
In the hiding-place of Jesus' Heart.
God the Spirit so hold us up
That we may drink of Jesus' Cup.

*C. Rossetti.*

### Echoes from the Outside World.

### Royalty at Whitsuntide.

The King and Queen passed their Whitsuntide at Windsor, leaving London on Saturday afternoon amid thunder and lightning. Fortunately the storm spent its violence whilst their Majesties were in the train, the weather upon their arrival being again beautifully fine. The King remained for some few minutes in conversation with the Mayor of Windsor, the Queen, who was attired in heliotrope and wore a bunch of roses, proceeding straight to the carriage with Princess Victoria. Within a few minutes of their arrival the King and Queen, having changed to riverside attire, went down to the banks of the Thames to see the new launch, which is as yet unchristened, and then inspected the skiff which has been built for their grandsons, Princes Edward and Albert of Wales. Amongst the guests staying at the Castle were Prince Andrew of Greece and his fiancée Princess Alice of Battenberg, together with her mother and father, sister and brother. A dinner party was given on Saturday evening for 22, at which the Prince and Princess of Wales—who with their five children were at Frogmore House for the holidays—were present. All the members of the Royal Family, including the two little boys, attended divine service in the private chapel on Sunday morning, and in the afternoon, notwithstanding that the Court was in residence, the King had commanded the East Terrace to be open to the public and the bands of the 1st Life Guards and the Scots Guards played alternately. They were also expressly bidden to play on Monday and Tuesday afternoons for the benefit of the holiday people.

### Serious Fire at Eton College.

Early on Monday morning a fatal fire occurred at Eton College, in the house of Mr. R. S. Kindersley, one of the masters. It was discovered about 4 o'clock, and an alarm was immediately raised. The flames, which broke out in the upper part of the building, made such rapid progress that the boys had great difficulty in clambering down from the windows, many making use of the thick stem of the wisteria which covered the house. The inmates consisted of Mr. Kindersley, his family, 31 pupils, and 11 servants. Two pupils were away, and the two boys who did not escape were Lionel Lawson and James Kenneth Horne. Both boys were about 14 years of age, and their bodies were recovered when the fire had been got under. Immediately the sad news reached Windsor Castle, the King and Queen, and also the Prince and Princess of Wales, sent sympathetic messages to the head master. The annual celebration of the Fourth of June was at once abandoned. The inquest was held on Tuesday, and the jury found that death was due to suffocation, and added a rider that the iron bars at the windows of some of the rooms should be removed. Shortly before one o'clock on Tuesday afternoon the Queen visited the scene of the fire, and having inspected the ruined buildings passed through the brick archway at the end of the chapel into the school quadrangle. Her Majesty had a long conversation with Mr. H. M. Bland, one of the assistant masters who gave evidence at the inquest.

### Russia and the "Times."

The *Times* correspondent in St. Petersburg, Mr. Barham, has been expelled from Russia by order of the Assistant Minister of the Interior, the reason given being his hostility to the Russian Government and the invention of false news. A police officer called upon him on May 28th and invited him to proceed to the police-station. There he was informed that he was under arrest, and he was forbidden to send a message to anyone, even to his wife or to the Ambassador. However, after a little time he was set at liberty, having entered into a written bond to leave Russia by the first train to the frontier. He then laid the facts before Sir Charles Scott, the British Ambassador, who succeeded in getting him allowed three days, instead of eight hours, in which to settle his affairs. The Minister of the Interior informed the Ambassador that no objection was entertained towards the *Times* correspondent personally, or to anything in particular that he had written, but that the tone of his correspondence and of the *Times* in general was regarded as hostile, that the Russian authorities had determined that he could no longer be tolerated in St. Petersburg, and that "they were resolved to give the *Times* a lesson."

### Women and Universities.

It has been decided by the council of Dublin University to approve of the scheme for the admission of women to all the rights and privileges of the University, including the conferring of degrees in arts, medicine, engineering, and music. Compulsory Greek is to be abolished. The senate will meet on Tuesday next, and, should their report be favourable, a King's letter authorising the necessary changes will be sought for. The opening of Trinity College to women has been practically forced upon the board by the great number of degrees conferred by the Royal University on female students within the last few years.

### Severe Thunderstorms.

London and the home counties were visited by exceptionally severe thunderstorms on Saturday afternoon and the early hours of Sunday morning. The special feature of the first storm was that for two hours the thunder and the lightning continued without a drop of rain falling and with the sun still shining in the sky, although the atmosphere was hot and stuffy. Then suddenly the clouds broke and the rain descended with tropical force. At Croydon four persons took refuge in the doorway of a half-finished house and they were all struck by lightning. Two were killed outright, the other two being only injured. At the inquest on the bodies the coroner said that he thought the manner in which the house had been struck was worthy of investigation by the Meteorological Society. In South London houses were flooded, and pleasure-seekers on the Thames were caught in the downpour and soaked through before they could even land for shelter. A labourer at Petersham had his clothes burned and two of his limbs paralysed. At Danesfield House, Medmenham, the lightning tore gaps in the leaded roof, so that the rain poured through and severely damaged some tapestries worth thousands of pounds. Isolated cases of persons killed or injured occurred in several places. The storm in the night, though more violent, caused no loss of life and comparatively little damage, though at Shortlands, in Kent, a large private house was set on fire. The local fire brigade quickly subdued the conflagration.

### The Walking Mania.

On Whit Monday the waitresses followed the example of the members of the Stock Exchange, and had a walking competition of their own. The start was made at half-past seven in the morning from the Royal Exchange, and the goal was the Marble Arch. A large number of young women, attired in every-day waitress garb, with white aprons and straw hats, completed the distance, and the first to arrive at the winning post, who covered it in 47 minutes, received a five-pound note. A few of the girls fainted or collapsed from hysteria on the way, and the public, of whom a great crowd collected, cheered or jeered according to their humour.

### A Book and its Story.

NEW NOVEL BY MRS. F. REYNOLDS.*

Mrs. Reynolds' latest novel is brightly, if slightly written, and one follows with a certain excitement the love story of the pathetic little heroine and the inscrutable hero from the first moment of their meeting to the close of the story. The other characters, not unordinary, are all fair specimens of the type to be met with in the summer season at country places where paying guests are taken. These are only necessary as diversions. The Little Teacher and The Man with the Wooden Face alone hold the reader. On the opening page is this description of the brave Brontë-like little person whom Mrs. Reynolds has chosen as her heroine. "She was only a little music teacher, not really old, but already somewhat worsted in the battle of life, having for years now, very long years they seemed to her, wrestled single-handed with the grim battalions of poverty. None but she knew how thin at times the line of defence had been, how at others ammunition had all but failed, or to how low an ebb, now and again, had the siege rations fallen. But in that camp surrender meant effacement under the feet of the cruel conqueror, and though, at times, the struggle seemed almost hopeless, the thin flag of respectability had never yet failed to flutter limply from the standard, and the gallant little defender still struggled on, working early and late, and taking scant rest at night." She had not always led this weary monotonous life; there were far-off memories of a happy, sheltered childhood seen through "an atmosphere of golden haze, with a country home, a [illegible] father, a dainty, winsome mother." But these visions passed, and the day came when, at the age when girls are looking forward to emancipation from schoolroom routine, there fell upon the Little Teacher the blow which had resulted in the loss of home and position. An added poignancy was given in the whispered rumours that it had been caused through some dishonourable action of her father. "These rumours she set on one side. There were things she did not *want* to know." To her no lover had come, but she was still just an imaginative child at heart, and wove visions of a possible one who might yet enter into her life, taking off its burden and lifting her high above its tiresome routine. The Little Teacher was no longer young. "For twenty years she had struggled with batch after batch of juvenile performers." In spite of her being no longer a girl, "she retained her girlish figure, surmounted by its small, well-poised head, around which the abundant brown hair was softly braided. The face below the hair was clear-complexioned, if somewhat pale . . . yet the pallor was not unhealthy; the somewhat thin lips were firm and red, despite the tired lines about them, and although there were dark rings below the grey eyes, the eyes themselves, clear and steadfast, looked out in a trusting way from the almost childlike face." When her day's teaching was done she relieved the tedium of her solitary evenings by entering as a competitor for some of the many prizes offered for the solution of more or less foolish puzzles. One day she was rewarded by seeing her name as the successful owner of a third share of the sum of money given by a leading paper for a list of the most popular pictures of the year. What to do with it had hardly entered into her mind. Someone suggested she should take a holiday, and "the more the Little Teacher thought of it the more delightful, the more practicable, the idea became." Holidays were things unknown to her since she became a working woman. An advertisement had constantly caught her eye of "Llanatro," a place in Wales possessing many attractions. Mountains, lakes, wooded glens, the sea coast, and a shell island. What could be more fascinating than the visions conjured up? Three weeks in such surroundings, far from the heat and noise of London in July, would be paradise indeed. And when the Little Teacher finds herself before long, at the end of her journey, this was the greeting that Llanatro gave to her. "A radiant evening with the sun already settling gently down in the rose scattered west. Long shadows of wind-slanted trees, great purple hollows in the gold touched mountains, the sweep and cry of birds sailing homewards across the marshes, the air filled with the salt, strong smell and taste of the unseen sea." There were other travellers too, as an overloaded waggonette, crowned with a bicycle and an inverted mail-cart testified, and the owners of golf clubs were bargaining for their conveyance with the driver, who with the aid of the solitary porter, was with difficulty securely balancing the miscellaneous collection of luggage on his vehicle. The Little Teacher in her limited life knew few people by name, but she was not without imagination and a quiet sense of humour, and had acquired a mental habit of giving pseudonyms, fitting their prevailing characteristics, to many of those whom she daily met, in passing to and fro from her work in town. Standing aside from the lively group was a man whose quiet bearing was conspicuous. "He was a tall, strongly-built man, clad in grey tweed . . . . When he at last turned round showing a face as immovable apparently as his recent attitude, the thought went through the Little Teacher's mind like a flash: "What a wooden face! and so it was. Tanned by exposure to hotter suns than ours, clean shaved, the somewhat high cheek bones, the grooved upper lip, the firmly modelled chin, might have been carved from wood. A not unhandsome, but save for the dark eyes under the overhanging brows, a singularly lifeless face." A few minutes later he took the vacant place opposite the Little Teacher in the waggonette. "So he came into her life." In silence they sat, for the Man with the Wooden Face did not speak until the Little Teacher, tired out with her journey, suddenly found herself sadly oppressed and filled with vague misgivings as to how her holiday would turn out, and "What could she say to those vague, formless beings as yet, whom she was about to meet. Perhaps it would have been better not to have come. The childlike mouth drooped a little, and the grey eyes filled with tears." But the Man with the Wooden Face, if he did not speak, apparently observed. "His companion was, of course, nothing to him. . . . . Yet at the moment when the tears threatened to brim over he bent forward, and for the first time opened his lips." "Travelling is so tiring," he said gently, "Will you allow me to arrange things a little more comfortably for you. . . ." She thanked him with a smile. "He smiled back, only, be it noted, with his eyes; the wooden mouth seemed quite incapable of the levity of a smile." To the Little Teacher nothing surely in the whole world could be more beautiful than the scenery through which they were passing. The wooded crags rose on either side of the winding river that leaped among its mossy boulders below. We leave the two here, at the beginning of their romance, with genuine regret, and cordially recommend our readers to obtain Miss Reynold's book and follow out for themselves the fortunes of her interesting lover and heroine.

* "The Man with the Wooden Face." By Mrs. F. Reynolds. (1 vol. 6s. Publishers, Hutchinson and Co.)

### Notes and Queries.

**REGULATIONS.**

**The Editor is always willing to answer in this column, without any fee, all reasonable questions, as soon as possible.**

**But the following rules must be carefully observed:—**

**1. Every communication must be accompanied by the name and address of the writer.**

**2. The question must always bear upon nursing, directly or indirectly.**

**If an answer is required by letter a fee of half a-crown must be enclosed with the note containing the inquiry. We cannot undertake to forward letters addressed to correspondents making inquiries unless a stamped envelope is enclosed.**

*Training for District Nurse.*

(83) Will you kindly tell me what should be the minimum qualification or training for a district nurse in a large suburban parish?—*Member of the Committee.*

The training required by Queen Victoria's Jubilee Institute for Nurses is two years in some approved hospital or infirmary, with training in district nursing for not less than six months.

*Indian Nursing Service.*

(84) To whom should I apply for particulars regarding the Indian Nursing Service? I have been in South Africa on the Army Reserve for two and a half years.—*M. C.*

Address Under Secretary for India, India Office, St. James's Park, S.W.

*Massage.*

(85) What is the shortest time in which I could gain a certificate in massage? Where could I learn? and what would the terms be?—*C. B. B. A.*

You can learn at the Hospital for Consumption, Brompton; the Hospital for Epilepsy and Paralysis, 32 Portland Terrace, Regent's Park, N.W.; the National Hospital for the Paralysed and Epileptic, Queen's Square, Bloomsbury, W.C.; the West End Hospital for Nervous Disorders, 78 Welbeck Street, W. (one year's training in general nursing, massage, and electricity is given free to accepted probationers in return for one year's service at the last-named hospital); or you can obtain reliable advice and hear of private teachers through the Secretary, the Society of Trained Masseuses, 12 Buckingham Street, Strand, W.C. The terms vary from £10 10s.

*Midwifery. Massage.*

(86) I have been hospital and private nursing for 10 years, but I have no certificate. Can you kindly tell me of some institution, private or otherwise, where I could be well taught massage and electricity and gain a certificate? Can I get taught free of charge if I give my services? If not too much trouble, will you kindly tell me where I could get free training in midwifery?—*A Ten Years' Nurse.*

See reply to *C. B. B. A.* From what you say you would probably do better with the midwifery. See advertisements or advertise in our columns. It is impossible to get free training in either massage or midwifery, yet with your long experience a private institution might be willing to give you the required instruction for services covering a suitable period.

*Manchester.*

(87) Will you kindly tell me if there is a Nurses' Co-operation in Manchester, and, if so, will you give the address?—*L. W.*

We have no particulars of such an institution.

*Rheumatism.*

(88) Will you kindly tell me what I can do to get rid of gout in my system? It is now in my feet and I am afraid that it is coming in my hands. I take a grain of calomel at night occasionally.—*Nurse H.*

We cannot prescribe. Put yourself under medical treatment at once, and do not take a powerful drug like calomel unless under medical guidance.

*Male Nurses.*

(89) Are there any hospitals in London which train male nurses?—*Masseur.*

The National Hospital for the Paralysed and Epileptic, Queen's Square, Bloomsbury, W.C., is the only one. Male nurses are trained in some of the infirmary wards of the larger asylums for the certificate of the Medico-Psychological Society. See "The Nursing Profession: How and Where to Train," for list of Asylums.

*Local Government Board Regulations.*

(90) Will you kindly tell me what qualifications nurses must hold to enable them to comply with the regulations of the Local Government Board. Would a thorough training in a large asylum be sufficient?—*F. W.*

In order to be qualified for the post of superintendent nurse under the Local Government Board, nurses must have a certificate of three years' training in general nursing from a school which is recognised by the Board. Such schools must maintain a resident medical officer. Asylum training would not be recognised.

*Hospital Training.*

(91) I shall be very glad if you will give me the name of any institution similar to the Nursing Sisters of St. John the Divine, in or near London, where ladies can receive training.—*G. F.*

Many hospitals offer facilities to paying probationers to take up a short course of training. See "The Nursing Profession: How and Where to Train." But we do not understand your question fully. Do yo mean a religious institution?

*Elementary Nursing.*

(92) Would anyone inform me what kind of lectures to give to a class of girls belonging to a Girls' Friendly Society? I am asked to speak on cleanliness, ventilation, and on general first aid. Is there any book that I could get to make it more simple to them?—*District Nurse.*

Write to the Secretary, the National Health Society, 53 Berners Street, London, W.

*Hospital Training.*

(93) I am now 17. Is it possible for me to enter a nursing home now, in order to ensure my being taken as probationer by a London hospital when I am of a proper age?—*Nörline.*

There would be no advantage in entering any nursing home now. You will not be eligible until you are 23.

*Guilds.*

(94) Will you kindly let me know the rule of the guilds of St. Barnabas and St. Veronica, and to whom application should be made?—*E. C. W.*

The Secretary, the Guild of St. Barnabas for Nurses, the Nurses' Hostel, Frances Street, W.C., and the Secretary, the Guild of St. Veronica for Trained Nurses, 1 Mardale Crescent, Edinburgh, will give you every information.

*Home.*

(95) Please tell me of a home or charity where a poor woman suffering from spastic paralysis, and who is helpless, could be received for 7s. 6d. weekly.—*E. M.*

Have you applied to the All-Hallows County Hospital, Ditchingham, Bungay, Norfolk?

Could you tell me of a home (Midlands preferred) where a gentleman of 73 could be received? He is invalided from paralysis, but is mentally healthy. His daughter could afford to pay 5s. a week towards his support.—*E. M.*

You will find a list of homes for chronic and incurable cases in "Burdett's Hospitals and Charities;" but none, we fear, willing to accept the patient you mention at 5s. a week.

*Hospital Training.*

(96) I am 21, tall and strong, and have always wished to become a nurse. Will you tell me if the fact that I have been a housemaid will prevent my being accepted as probationer in the best hospitals? I do not wish to enter a workhouse infirmary.—*C. E. M.*

You are still too young to be eligible for training in the best general hospitals. When you are 23 you might write to the Matron of the Middlesex Hospital, Mortimer Street, W.

*Dispensing.*

(97) I am in a small workhouse infirmary where a knowledge of dispensing would be useful. Can you recommend me a simple book explaining the properties of drugs, and their abbreviations, etc.?—*Irishwoman.*

Probably "Notes on Pharmacy and Dispensing for Nurses" (published by the Scientific Press, 28 & 29 Southampton Street, Strand) would help you. But you cannot learn dispensing by means of a book only.

*Home School.*

(98) Can you tell me of a school where a boy of eight, suffering from partial paralysis of hearing and sight, could receive education and training? Parents could pay something for his maintenance.—*C. Z.*

Write to Mrs. Burgwin, of the London School Board, 21 Claylands Road, Clapham, S.W., or Miss Mabel Anderson, Chute Manor, Andover, Hants.

**Standard Nursing Manuals.**

"The Nursing Profession: How and Where to Train." 2s. net; 2s. 4d. post free.

"Nursing: Its Theory and Practice." (Revised Edition). 3s. 6d. post free.

"Surgical Ward Work and Nursing." (Revised Edition). 3s. 6d. net.; 3s. 10d. post free.

"Practical Handbook of Midwifery." (New Edition). 6s. net; 6s. 3d. post free.

"Notes on Pharmacy and Dispensing for Nurses." 1s. post free.

"Fevers and Infectious Diseases." 1s. post free.

"The Art of Massage." (New Edition). 6s. post free.

## Figures and Tables

**Figure f1:**